# Online Measurement of Real-Time Cytotoxic Responses Induced by Multi-Component Matrices, such as Natural Products, through Electric Cell-Substrate Impedance Sensing (ECIS)

**DOI:** 10.3390/ijms161126014

**Published:** 2015-11-12

**Authors:** Adyary Fallarero, Ana E. Batista-González, Anna K. Hiltunen, Jaana Liimatainen, Maarit Karonen, Pia M. Vuorela

**Affiliations:** 1 Discipline of Pharmaceutical Biology, Division of Pharmaceutical Biosciences, Faculty of Pharmacy, University of Helsinki, 00014 Helsinki, Finland; anna.k.hiltunen@helsinki.fi (A.K.H.); pia.vuorela@helsinki.fi (P.M.V.); 2 Department of Microbiology and Immunology, Albert Einstein College of Medicine, Yeshiva University, Bronx, NY 10461, USA; ana.batista@einstein.yu.edu; 3 Laboratory of Organic Chemistry and Chemical Biology, Department of Chemistry, University of Turku, 20014 Turku, Finland; jh.liimatainen@gmail.com (J.L.); maarit.karonen@utu.fi (M.K.)

**Keywords:** ECIS, cytotoxicity, impedance, label-free, natural extracts, screening

## Abstract

Natural products are complex matrices of compounds that are prone to interfere with the label-dependent methods that are typically used for cytotoxicity screenings. Here, we developed a label-free Electric Cell-substrate Impedance Sensing (ECIS)-based cytotoxicity assay that can be applied in the assessment of the cytotoxicity of natural extracts. The conditions to measure the impedance using ECIS were first optimized in mice immortalized hypothalamic neurons GT1-7 cells. The performance of four natural extracts when tested using three conventional cytotoxicity assays in GT1-7 cells, was studied. *Betula pendula* (silver birch tree) was found to interfere with all of the cytotoxicity assays in which labels were applied. The silver birch extract was also proven to be cytotoxic and, thus, served as a proof-of-concept for the use of ECIS. The extract was fractionated and the ECIS method permitted the distinction of specific kinetic patterns of cytotoxicity on the fractions as well as the extract’s pure constituents. This study offers evidence that ECIS is an excellent tool for real-time monitoring of the cytotoxicity of complex extracts that are difficult to work with using conventional (label-based) assays. Altogether, it offers a very suitable cytotoxicity-screening assay making the work with natural products less challenging within the drug discovery workflow.

## 1. Introduction

Natural products have provided milestone drugs with highly-successful roles in modern pharmacotherapy. Their impact in many therapeutic areas is undeniable [1]. For instance, anti-bacterial drugs of natural origin account for more than 65% of all anti-bacterial drugs approved over the last 30 years [2]. In addition to their roles as drug leads, natural products also provide highly-diverse starting scaffolds for structural optimization and serve as chemical tools for validating new molecular targets [3]. Over the past decades, the analysis, separation, and structural elucidation of natural products have greatly advanced, but tracking the bioactivity—including cytotoxicity—remains a key issue hampering a larger exploitation of natural products in drug discovery. Their chemical complexity causes various interferences with the performance of label-dependent methods [4]. A well-known example is the alcoholic extract of *Hypericum perforatum*, which contains as one of the major components an anthraquinone derivative known as hypericin, that displays a strong autofluorescence signal with an emission maximum close to that of rhodamine red dye [5]. In addition, natural compounds such as luteolin, quercetin, kaempferol, and resveratrol—widely present in natural sources and foodstuffs—can interfere with redox probes used in cell viability assays, *i.e.*, tetrazolium dyes [6,7,8]. These elements support the need for continuous development of bioactivity tracking methods that can be applied without interference from the constituents of natural extracts. Especially, the assessment of the cytotoxicity is an important step in the discovery of new drugs [9], as clinical trials are haunted by a very low success rate with 20% of the rejections associated to unanticipated toxicity [10].

Label-dependent methods have been exhaustively used in the assessment of the cell viability during predictive toxicological screens. This popularity is associated to their high-throughput capacity, relatively low costs, and robustness in their responses. Nonetheless, these methods have various disadvantages, such as the disturbances that can be caused by the added labels and the interference that can be generated by autofluorescent or colored samples, which can all lead to false results [11]. Label-free methods are able to circumvent those problems by focusing on cellular responses that can be measured in the absence of chemical labels and without interference from colored and autofluorescent samples. They allow non-invasive, continuous readouts that offer a simultaneous view of acute and long-term cytotoxicity events with minimal labor involved. Due to this, label-free methods have been increasingly regarded as powerful alternatives that can be utilized during cytotoxicity screenings [4], and have also been exploited in the cytotoxicity assessment of natural products [12,13,14].

Electric Cell-substrate Impedance Sensing (ECIS) is a label-free technology based on the measurement of the impedance changes taking place when an alternating current (AC) is applied on cells that are seeded onto a pair of small gold electrodes. In a typical ECIS experiment, cells are added into electrolyte (culture medium)-filled dishes having the gold electrodes on the bottom. An oscillator applies an AC signal of amplitude 1 V through a series 1 MΩ resistor to the two-electrode system. The current flow in the system remains constant at 1 µA. After the cells are added, they drift and start attaching into the electrode. Due to the membrane capacitance, the attached cells block the current flow, forcing the current to trespass along narrow electrolyte-filled channels between cells. This results in detectable increases in impedance. Thus, by recording those impedance changes, the attaching and spreading of the cells can be monitored. Additionally, morphology changes of cells (such as apoptosis and necrosis) can also be detected. These changes cause the impedance to decrease, since the current flows more freely due to damaged (necrosis) and shrunk (apoptosis) cells [15]. This method was first described by Giaever and Keese [16] to measure changes in the morphology of fibroblasts. Initially, the acceptance of this technology as a tool in drug discovery evolved at a slow pace, but over the past 10–15 years, it has increasingly attracted interest. ECIS has been applied to follow apoptosis [17], to monitor cell viral infection [18], in wound-healing assays [19], as well as to measure cytotoxicity [20,21,22,23,24] and cytoprotective effects [15]. In comparison to classic acute cytotoxicity assays, ECIS has been shown to produce similar endpoint results [22], which adds up to the ECIS’s ability to monitor continuous cytotoxic responses.

Within the natural products research field, ECIS has been utilized to investigate various biological activities, e.g., their angiogenic [25], neuroprotective [15], anti-tumor actions [26,27,28], as well as their effect in ocular diseases [29]. However, in spite of these potentially significant benefits, the application of this technology on the cytotoxicity study of complex samples mixtures, such as crude natural extracts or fractions, has not been attempted. Thus, the aim of this paper was to assess the applicability of the label-free technology ECIS in cytotoxicity studies of natural products and to critically examine the possible advantages of this method to overcome some of the limitations of conventional assays. The cytotoxicity of the silver birch extract, which was found to interfere with three conventional assays, was studied in GT1-7 (mice immortalized hypothalamic neurons) using ECIS.

## 2. Results and Discussion

### 2.1. Optimization of the Cell-Substrate Impedance Sensing (ECIS) Assay

The ECIS method was optimized to use with a model cell line, GT1-7 mice immortalized hypothalamic neurons, which had not been previously utilized in ECIS. Our group has earlier reported the usefulness of GT1-7 cells in cytotoxicity screenings of large collections of plant extracts [30] and, beyond our own studies, GT1-7 cells have been widely used in cytoprotective studies [31,32,33]. Different cellular concentrations and electrode types were first compared (Table 1). The best statistical parameters were obtained in uncoated 8W1E electrodes seeded with the highest cellular concentration (4 × 10^5^ cells/mL). In these conditions, the S/B ratio (signal-to-background; ratio between the maximal and minimal signals) at 24 and 48 h was larger for 8W1E electrodes, and the variations of signals were lower, resulting in *Z′*-values (screening window coefficient) of 0.92 at 24 h and 0.89 at 48 h. These high *Z′*-values obtained here, well over 0.5, indicate that the assay is very robust in providing a clear-cut distinction between toxic and non-toxic responses. In cell-based assays, *Z′*-values are typically lower than in biochemical assays, due to the larger signal variations characteristic of cell-based systems [34].

**Table 1:** Summary of the statistical parameters: screening window coefficient (*Z′*), signal-to-noise (S/N) and signal-to-background (S/B), measured during the optimization assays conducted in uncoated 8W1E and 8W10E electrodes.

Cell Concentration (Cells/mL) × 10^5^	Time (h)	8W10E			8W1E		
		*Z′*	S/N	S/B	*Z′*	S/N	S/B
4	24	0.53	6.88	1.88	0.92	44.13	4.09
	48	0.56	7.36	2.52	0.89	38.00	3.92
2	24	0.11	2.81	1.86	0.63	10.11	3.24
	48	0.16	3.74	2.17	0.26	4.46	3.56
1	24	0.17	4.05	1.34	0.42	2.38	2.23
	48	0.23	4.36	1.53	0.04	3.48	2.93

**Figure 1:**
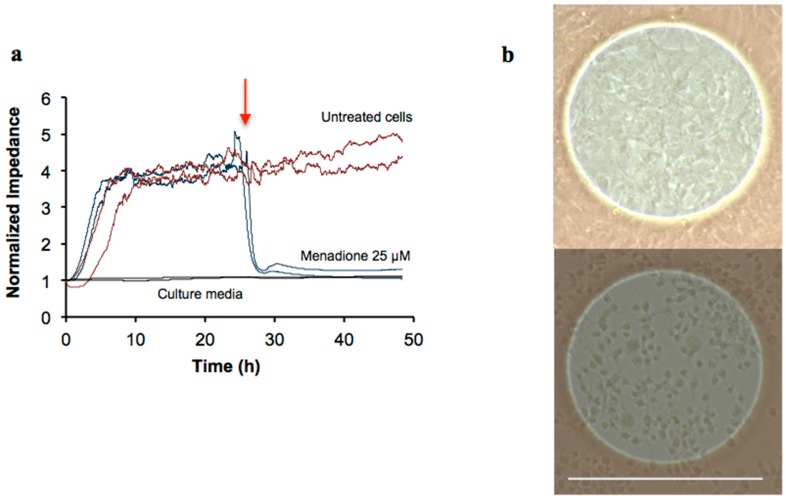
(**a**) Impedance signal recorded in untreated cells (4 × 10^5^ cells/mL) added to uncoated electrodes for up to 48 h, or in cells exposed to menadione (25 µM) after 24 h of initiating the experiment or in culture media. The red arrow indicates the time point where menadione was added; and (**b**) transmitted light microscopy pictures taken of the untreated (**up**) and menadione-treated cells (**bottom**) after 48 h. Scale bar represents 0.25 mm, for both images.

A typical behavior of replicated samples in which cellular suspensions (4 × 10^5^ cells/mL) were inoculated into uncoated 8W1E electrodes, is shown in Figure 1a. ECIS curves are characterized by three distinctive phases. Firstly, the so-called lag phase occurs in which only a very small increase in impedance is detected, as the cells need time to attach to the electrode. This is followed by the attachment and spreading phase that is associated with the coverage of the electrodes and it is reflected by a linear increase in impedance. Finally, the third phase takes place when the electrodes are fully covered and an impedance plateau is reached. In this stage, some fluctuations can still be detected, that have been associated to cellular micromotions, based on the early studies of Giaever and Keese [35], in which cells were fixed with formalin, resulting in a disappearance of these fluctuations.

Cells (4 × 10^5^ cells/mL) seeded in uncoated 8W1E electrodes quickly attached and spread over the electrodes, reaching the plateau phase within the first 10 h. After 24 h, these samples were well stabilized in the plateau phase. The coefficient of variations of the maximal and minimal signal, calculated from four replicate wells in plate-to-plate or day-to-day basis, was lower than 10% when measured at 24 h and lower than 9% at 48 h. Thus, maximal and minimal signals, measured on the plateau phase, were not only sharply distinct, but also stable between ECIS traces, thus ensuring a higher degree of certainty when performing single-point screens.

Another factor to consider during assay optimization is the time effectiveness. The ECIS curves (Figure 1a) showed that a fast attachment and spreading phase took place, but uncoated electrodes were only fully covered after 10 h. As a strategy to shorten the assay time, protein coatings of the electrodes with fibrinogen and fibronectin were tried out (BSA coating was used as control). Coating with bovine serum albumin (BSA) or fibrinogen significantly slowed down the attachment of the cells, in comparison with uncoated electrodes. In contrast, in fibronectin-treated electrodes, the kinetic of the attachment of the cells improved as reflected in an increase in the slope of the attachment phase. Similar results were obtained by [36] using epithelial canine kidney cells (MDCK strain II), which spread significantly faster in fibronectin-coated electrodes in comparison to any other protein coatings these authors examined.

However, in fibronectin-coated and uncoated electrodes the cells reached the plateau phase at time points that, from a practical perspective, were not significantly different. Moreover, after 24 h, the uncoated electrodes provided better quality parameters (Table 1) when compared to fibronectin-coated electrodes (*Z′* = 0.76; S/N = 15.25; S/B = 3.01). Thus, uncoated electrodes were deemed the best practical choice.

The integrity of the GT1-7 cells on the ECIS electrodes after a 48-h incubation period was authenticated by additional imaging tests. AFM was first used to scan the surface of uncoated electrodes covered with 4 × 10^5^ cells/mL or culture media (Figure S1a). The result showed that the thickness of the cell monolayer was roughly 500 nm, while the diameters of the neurons fall within the micrometer range (spanning from 5 to 15 µm). The viability of the cells attached after 48 h was further followed-up using Fluorescence Microscopy (Figure S1b). The predominance of the green fluorescence (due to calcein staining of metabolically-active cells) over the red fluorescence (indicative of ethidium homodimer-1, EthD-1, stained cells with damaged membranes) demonstrated that the high impedance values registered in the ECIS trials are indeed associated to predominantly-living cells.

To emulate cytotoxic effects, a model neurotoxicant was added; in this case menadione. The acute cytotoxicity of menadione at 25 µM was detected as a drop of the impedance values occurring right after the compound addition, 24 h after the seeding of the cells took place (Figure 1a). Transmitted light imaging confirmed that the confluent cellular monolayer (Figure 1b, top) was disrupted in menadione-exposed samples (Figure 1b, bottom) and fewer cells were left in comparison with the untreated controls. Those left after treatment also displayed a more rounded morphology with no clearly defined axons (Figure 1b, bottom).

### 2.2. Cytotoxicity Profiling of Four Natural Products Using the ECIS Assay

Traditionally, the cytotoxic assessment of natural products has been performed using label-based assays. A number of cytotoxicity methods are available that measure the damage of the membrane (*i.e.*, lactate dehydrogenase, LDH release), the cellular content of a key metabolite (*i.e.*, ATP (adenosine triphosphate) luminescent determination) or a dye reduction by metabolically-active (viable) cells (*i.e.*, 3-(4,5-dimethylthiazol-2-yl)-2,5-diphenyltetrazolium bromide, (MTT) and resazurin reduction) [37]. The occurrence of possible interferences due to natural extracts during the performance of these label-based assays was tested. For that purpose, four different extracts were chosen (coded NP1–NP4). NP1 is a natural extract obtained from *Betula pendula*, a widely spread tree in Finland, which has been used in folk medicine as a diuretic and anti-inflammatory preparation [38,39]. Three standardized commercial extracts (NP2–NP4) were also tested, obtained from *Silybum marianum* Gaertn (milk thistle), *Olea europaea* L. (olive), and propolis, respectively. They were selected based on the fact that they are commercial preparations that are sold in connection to indications as alternative medicines in the treatment of various diseases. The milk thistle extract (NP2) has antioxidative and oxidative stress-related injury inhibiting properties [40,41], and is recommended to alleviate hepatic diseases and intoxications [42]. The olive extract (NP3) is a natural supplement with cholesterol and blood pressure lowering properties [43]. Additionally it has *in vitro* antioxidative effects, and has been used as neuroprotectant in lead-induced neurotoxicity in rats, without described cytotoxic effects [44]. Propolis (NP4) is a resinous substance composed by sap, bark, and bee excreta, accumulated in bee hives. It is widely used as a health supplement with various claimed biological activities [45], such as antimicrobial, antioxidant [46], and neuroprotective effects [45,47].

Interference with the resazurin reduction method, an ATP-quantification (luminescent-based) cell viability assay, and the commercial LIVE/DEAD viability/cytotoxicity assay, were studied. For this purpose, the four extracts were incubated, in the absence of cells, with the three different probe systems (Table 2) and the conditions of a cellular assay were emulated.

**Table 2:** Optical readouts caused by birch (NP1), milk thistle (NP2), olive (NP3), and propolis (NP4) extracts using three cell viability assays, in the absence of cells. Values are shown as mean ± SD (*n* = 3).

Sample (Extract)	Resazurin Reduction Assay (RFU)	ATP Luminometric Assay (RLU)	LIVE/DEAD Fluorescence Assay (RFU)	
			Calcein	EthD-1
Probe control	83.8 ± 4.9	137.4 ± 4.6	6.671 ± 0.074	0.121 ± 0.017
NP1	176.6 ± 6.6 ***	1758.9 ± 65.3 ***	10.200 ± 0.092 ***	0.433 ± 0.013 ***
NP2	106.8 ± 2.9	119.4 ± 1.6	6.581 ± 0.112	0.151 ± 0.009
NP3	102.7 ± 28.8	122.0 ± 4.8	7.131 ± 0.134 *	0.143 ± 0.008
NP4	93.8 ± 6.6	122.0 ± 4.8	6.754 ± 0.102	0.190 ± 0.013 *

*** *p* < 0.0001, when compared to probe control samples; * *p* < 0.05, when compared to probe control samples; RFU and RLU indicate relative fluorescence units and relative light units, respectively; ATP corresponds to adenosine triphosphate and EthD-1 corresponds to ethidium homodimer-1; NP1-4 are defined in the title of this table.

Resazurin is a redox probe that permeates cells and becomes reduced to the fluorescent resorufin by mitochondrial, cytosolic, and microsomal enzymes from viable cells. The luminescent cell viability assay is aimed to quantify ATP content in living cells, based on the oxygenation of luciferin, a reaction that requires ATP and Mg^2+^, and is catalyzed by luciferase. The LIVE/DEAD viability/cytotoxicity kit uses calcein and EthD-1 to selectively stain live and dead cells. Calcein is a polyanionic dye that permeates live cells and become fluorescent upon the action of intracellular esterases. EthD-1 is able to enter cells with damaged membranes and bind to DNA resulting in a detectable fluorescent signal.

Sample NP2 did not significantly interfere with any of the probes used, while samples NP3 and NP4 were found to significantly interfere with the LIVE/DEAD viability/cytotoxicity kit, by significantly increasing the fluorescence of calcein and EthD-1, respectively. However, the most striking results were registered with NP1, which caused significant interferences in all assays. NP1 reduced the resazurin in the absence of cells, likely due to redox-active constituents. It also increased the signal of calcein and EthD-1 in the absence of cells, which could, to a certain extent, be explained on the autofluorescence of NP1. At the calcein emission fluorescence, NP1 has an autofluorescence of 2.31 ± 0.05, while at the EthD-1 emission fluorescence it has an autofluorescence of 0.14 ± 0.02. In a cytotoxic assay where the readout signal is proportional to the number of viable cells, NP1 can thus cause an overestimation of the cell viability, leading to false negative results.

**Figure 2:**
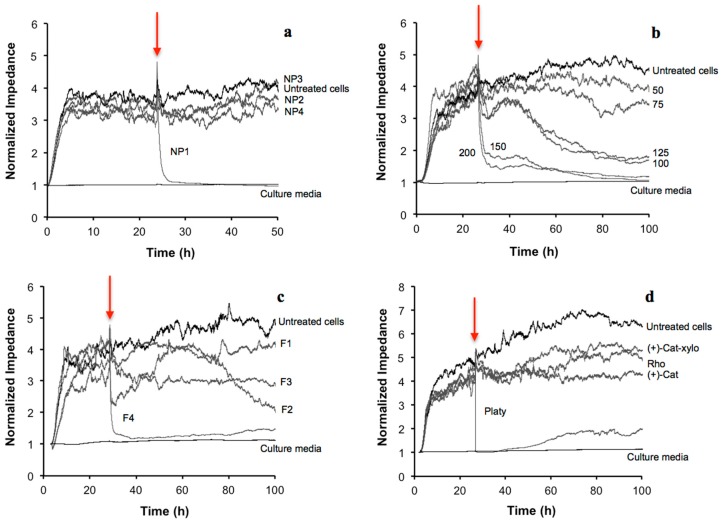
Impedance changes recorded with ECIS in cells treated with (**a**) birch (NP1), milk thistle (NP2), olive (NP3), and propolis (NP4) extracts (all at a concentration of 0.5 mg/mL); (**b**) birch extract, NP1 (at a concentration range of 50–200 μg/mL); (**c**) fractions F1, F2, F3 and F4 from NP1 (50 µg/mL) (see Experimental Section); and (**d**) pure compounds present on the fractions F1, F2 and F3 of NP1 at fraction concentrations of 50 µg/mL (platyphylloside 65.6 µM; rhododendrin 26.3 µM; (+)-catechin 7-*O*-β-d-xylopyranoside 24.7 µM; and (+)-catechin 35.9 µM). The red arrows indicate the time points where the samples were added for cytotoxicity assessments. Abbreviations used in the figure and legend: (+)-Cat corresponds to (+)-catechin; (+)-Cat-xylo corresponds to (+)-catechin 7-*O*-β-d-xylopyranoside; Rho corresponds to rhododendrin and Platy corresponds to platyphylloside.

Next, to avoid the occurrence of the interferences described earlier, the cytotoxicity of NP1–NP4 (all at a concentration of 0.5 mg/mL) was evaluated using ECIS (Figure 2a). The birch extract (NP1) was toxic to GT1-7 cells, distinguishable as a rapid drop to minimal impedance values that took place right after adding NP1. This extract seemed to cause a complete detachment of cells from the electrodes. On the contrary, the rest of the natural extracts (NP2–NP4) showed non-toxic effects, judging by the unaltered impedance kinetics.

### 2.3. Detailed Cytotoxicity Study of B. pendula Extract (NP1) Using the ECIS Assay

The cytotoxicity of the birch extract (NP1) was then subjected to a more detailed tracking using ECIS (Figure 2b). The extract was added 24 h after seeding the cells, as in previous experiments. The impedance curves showed an overall concentration- and time-dependent toxic effect of this extract. As expected, lower concentrations (50 and 75 μg/mL) were the least cytotoxic, while the higher concentrations (150 and 200 μg/mL) cause the most acute effects as the impedance decreased to around 50% of impedance values, compared to untreated cells, measured during the first 27 h after starting the experiments.

On the other hand, after adding middle-range concentrations of the NP1 (*i.e.*, 100 μg/mL), a sudden decrease of around 23% of impedance values compared to untreated cells was detected, after 30 h of starting the experiment. However, within a few hours, a recovery of the cells was recorded and after 40 h only a 9% decrease of impedance values was detected, compared to untreated cells. This suggested that early toxic events triggered by NP1 were temporarily controlled or buffered by the cells. Continuous monitoring of later changes in ECIS signal after 48 h also revealed that exposure to the middle-range concentrations (100 and 125 μg/mL) caused a steady impedance drop (over 50% decrease) at 72 h and later, indicative of delayed cytotoxicity. The elucidation of these specific biochemical events is a challenging problem given the complex chemical composition the extracts. However, in both of the cases described above, the effects would have been overlooked if the measurements had been conducted using only single-point measurements (for instance after 24 or 48 h), as in typical label-based assays.

To track the cytotoxic effect of NP1, a chemical fractionation was conducted, which resulted in four major fractions (F1–F4) with different chemical compositions, as presented in Table 3. Rhododendrin was present exclusively in F1, while platyphylloside was present in F1 and F2, with the highest amount found in F2. (+)-Catechin and (+)-catechin 7-*O*-β-d-xylopyranoside were present in all fractions except in F4. All fractions contained procyanidins in varying amounts but the largest amount was found in F4, which consisted exclusively of procyanidins. The dominant degrees of polymerization of procyanidins in fractions F1–F4 were 12, three, four, and seven, respectively.

**Table 3:** Chemical compositions of the birch extract (NP1) and its fractions (coded F1–F4). The results are expressed as mg of compound/mg dry fraction or extract ± SD (*n* = 6 for procyanidins and *n* = 3 for other compounds).

Sample (Extract or Fraction)	(+)-Catechin	Rhododendrin	Platyphylloside	Procyanidins
	(+)-Catechin 7-*O*-β-d-Xylopyranoside			
Crude extract	0.0581 ± 0.0018	0.0794 ± 0.0010	0.210 ± 0.010	0.28 ± 0.05
F1	0.00446 ± 0.00010	0.169 ± 0.007	0.142 ± 0.005	0.17 ± 0.03
F2	0.195 ± 0.006	–	0.533 ± 0.018	0.014 ± 0.007
F3	0.050 ± 0.004	–	–	0.51 ± 0.07
F4	–	–	–	1.00 ± 0.08

In the cytotoxicity evaluation of these four fractions with ECIS (all tested at 50 μg/mL), the method was able to distinguish distinct kinetic profiles (Figure 2c). When the cells were exposed to F1, they experienced a decrease of the impedance values, followed by a progressive, albeit not complete, recovery. With a slight shift on time, cells exposed to F3 were found to react similarly as those exposed to F1. A totally different pattern of response was seen in cells insulted with F2. In this case, no impedance changes were detected until around 70 h, but after this time the recorded impedance started to significantly decrease. Finally, fraction F4 was the most toxic, displaying an acute cytotoxic effect that was detected right after starting the exposure.

The high cytotoxicity detected in F4 can be explained on the basis of its chemical composition, as F4 exclusively contains procyanidins at high concentrations and high degree of polymerization (Table 3). It has been reported that highly polymerized procyanidins cause a high cytotoxicity [48], and the cytotoxic effect increases with the degree of polymerization [49]. The rest of the fractions contained lower amounts of procyanidins and other compounds in various amounts.

To clarify the cytotoxic effects seen with F1–F3, their main pure compounds were analyzed with ECIS (Figure 2d). Platyphylloside, rhododendrin, (+)-catechin and (+)-catechin 7-*O*-β-d-xylopyranoside were the main constituents of fractions F1–F3, and they were tested at the highest concentration present in the fractions (fraction concentrations of 50 µg/mL). Platyphylloside was found to be cytotoxic, while no impedance decreases were detected in the presence of any of the other compounds for up to 100 h of measurement. The absence of toxic effects when testing rhododendrin, (+)-catechin, and (+)-catechin 7-*O*-β-d-xylopyranoside concomitantly was also demonstrated (data not shown). Fraction F2 contained the highest amount of platyphylloside but the cytotoxic effects caused by F2 only appeared in the long-term (Figure 2c), in contrast to platyphylloside, which caused an acute cytotoxicity (Figure 2d). Thus, it cannot be ensured that platyphylloside could be the sole responsible molecule for the cytotoxic effects observed in F2. In our study, the cytotoxicity of platyphylloside was detected at 65.6 µM (the highest concentration present in the fractions at 50 µg/mL). This compound has been found cytotoxic with IC_50_ values around 10 µM, while IC_50_ values for (+)-catechin and (+)-catechin 7-*O*-β-d-xylopyranoside have been found higher than 100 µM in cancer, as well as non-cancerous cell lines [50], in accordance to our data.

Throughout the cytotoxicity tracking of the birch extract, a single technology, ECIS, was applied, and no need of adjusting assay conditions was encountered to avoid interference from the different samples that were generated in the chemical fractionations.

## 3. Experimental Section

### 3.1. Betula Pendula Extract

*Betula pendula* Roth (silver birch) bark material was obtained from a tree in the Botanical Garden of the University of Turku, Finland. The inner bark was cut, lyophilized, and ground in a mortar. The bark material was extracted with aqueous methanol [51]. The composition of the *Betula pendula* crude extract, coded as NP1, is available in [51]. Briefly, the extract contains high amounts of flavonoids, arylbutanoids, diarylheptanoids, simple phenolic compounds, phenolic acids, lignans, and procyanidins. The stock solutions of NP1 and its fractions were prepared in DMSO (dimethyl sulfoxide) at 100 mg/mL. NP1 (5.0 g) was fractionated using Sephadex LH-20 column chromatography (GE Healthcare Life Sciences, Helsinki, Finland) into four fractions (coded F1–F4) by elution with 20%, 40%, and 60% ethanol and, finally, with 70% acetone, respectively. The main components: platyphylloside, rhododendrin, (+)-catechin, and (+)-catechin 7-*O*-β-d-xylopyranoside, were identified by HPLC-DAD-HR-ESI-MS and quantified by HPLC-DAD [51]. For quantitation and cytotoxicity assays, platyphylloside, rhododendron, and (+)-catechin 7-*O*-β-D-xylopiranoside were isolated as in [51], with a purity of 94%, 91%, and 95%, respectively, while (+)-catechin was purchased from Sigma (St. Louis, MO, USA). Procyanidin contents in NP1 and its fractions were determined by HCl-butanol assay [52]. The procyanidin standard was isolated from NP1 by Sephadex LH-20 column chromatography and the dominant degree of polymerization of procyanidins in each fraction was determined by hydrophilic interaction HPLC-DAD-HR-ESI-MS [53]. Stock solutions of the pure compounds were prepared in DMSO at 20 mM, while NP1 and its fractions were prepared in DMSO at 100 mg/mL. They were stored at −20 °C until use.

### 3.2. Commercially-Standardized Natural Extracts

Commercially-standardized extracts obtained from *Silybum marianum* Gaertn (milk thistle), *Olea europaea* L. (olive), and propolis were purchased from Soria Natural (Soria, Spain). They were coded NP2–NP4, respectively. The main components in the milk thistle (NP2) and olive (NP3) extracts are silybin and oleuropein, respectively, both present at 6 mg/mL in the extracts, whereas propolis extract (NP4) is sold as a mixture of phenolic compounds (5 mg/mL), including caffeic acid, luteolin, and chlorogenic acid. NP2 and NP3 are sold as commercial preparations in glycerine (extract concentrations of 1700, 1200 mg/mL respectively) while NP4 is sold in propylenglycol at 5000 mg/mL. All extracts were stored at room temperature, following the instructions given by the manufacturer.

### 3.3. Cell Culture Conditions

Mouse immortalized hypothalamic GT1-7 cells [54] were cultured in Dulbeco’s Modified Eagle medium (DMEM) medium, supplemented with 10% fetal bovine serum (FBS), 50 µg/mL streptomycin, and 50 IU/mL penicillin, as in [55]. The cells were cultured in 75 cm^2^ cell culture flasks to 90%–100% confluence and harvested by adding a solution containing 0.05% (*w*/*v*) trypsin and 0.02% (*w*/*v*) EDTA (ethylenediaminetetraacetic acid) in PBS (phosphate-buffered saline). For the ECIS studies, cells were diluted to achieve the desired cellular concentration.

### 3.4. Impedance Measurements with ECIS

For impedance measurements, an ECIS instrumentation model *Z* (Applied Biophysics, Troy, NY, USA) was used. Electrodes were pre-treated with an aqueous 10 mM cysteine solution as recommended by the manufacturers. To start the trials, the electrodes were filled with 400 µL of DMEM. Several stabilizations steps were performed until the average and the variance of the impedance computed for all wells reached a plateau of around 2500 and 500 ohm, respectively. Then, the impedance was recorded at 16 kHz every 60 s and the signal was allowed to stabilize for 1 h. After the stabilization period, cells (400 µL) were added and impedance values continued to be measured in the same conditions. 24 h after adding the cells, 40 µL of the medium was replaced by 40 µL of the tested samples or corresponding volume of fresh medium in the untreated control wells. Solvent controls (DMSO, glycerol) were included at concentrations below 0.5%.

### 3.5. ECIS Optimization Trials and Repeatability

Two types of standard eight-well arrays (8W1E and 8W10E) were tried out, along with different cell concentrations (1 × 10^5^; 2 × 10^5^, and 4 × 10^5^ cells/mL). The 8W1E array has one active electrode in every well, while the 8W10E has ten active electrodes. The size of one active electrode is 250 μm in diameter, which means that around 50 cells with a cellular concentration of 10^5^ cells/cm^2^ will be measured on the active electrode. Moreover, the effect of two coating agents (fibronectin and fibrinogen) was assessed using BSA as the negative control. Coating protocols were followed as in [36]. Briefly, 100 µL of the protein solution (100 µg/mL in sterile water) was added to the electrodes, and incubated for 75 min at room temperature, followed by two washings with sterile water. After coatings, the ECIS measurements were conducted as described in the Section 3.4 before. Once the optimal conditions were established, repeatability studies were performed using at least two wells in the same trial (well-to-well variations). Plate-to-plate and day-to-day variations were measured with at least four wells. In these trials, samples corresponding to maximal (untreated cells) and minimal (culture medium) signals were included, as described in Section 3.9. Abolishment of the ECIS signal was induced with a model neurotoxicant (menadione) at a final concentration of 25 µM.

### 3.6. ECIS Signal Authentication by Imaging

Atomic Force Microscopy (AFM) was used for imaging the cell layer on the ECIS electrodes. Samples corresponding to maximal and minimal signals were tested, as defined in Section 3.9. Briefly, electrode pieces were cut out from the ECIS dishes and they were placed in a six-well plate and flooded with 5 mL of 4 × 10^5^ cells/mL or culture medium. Pictures were taken at 48 h after the cellular addition. Images were obtained using a Nanoscope IIIa scanning probe microscope equipped with a J-scanner (Digital Instruments Inc., Santa Barbara, CA, USA). The fluorescence staining was done directly onto the ECIS electrodes. As before, maximal and minimal signal wells were included. The LIVE/DEAD viability/cytotoxicity kit (Molecular Probes, Invitrogen, Carlsbad, CA, USA) was used, which is based on the detection of membrane integrity with EthD-1 (stains dead cells) and enzymatic activity with calcein (stains metabolically active cells). Cells (4 × 10^5^ cells/mL) were grown in uncoated ECIS electrodes for 48 h. Media was replaced by 200 µL of PBS containing calcein (2 µM) and EthD-1 (4 µM) followed by 45 min incubation at room temperature. Pictures were obtained with a Zeiss AxioVert 200M microscope (Carl Zeiss MicroImaging, Jena, Germany), using FITC and TRITC filters. Furthermore, untreated (4 × 10^5^ cells/mL) and menadione-treated cells were imaged after 48 h on the ECIS electrodes using an EVOS transmitted light microscope (Advance Microscope Group, Botwell, WA, USA).

### 3.7. ECIS Cytotoxicity Studies with Natural Products

ECIS was used to assess the cytotoxicity of NP1–NP4. GT1-7 cells were seeded at 4 × 10^5^ cells/mL in the electrodes and 24 h later, each extract (0.5 mg/mL, in DMEM) was added. Fractions of NP1 (F1–F4) were prepared in DMEM and assessed with ECIS at 50 µg/mL in a similar fashion. Pure compounds present in each fraction (50 µg/mL) were also tested with ECIS at the following concentrations that represent the highest concentrations of the compounds in the fractions: platyphylloside 65.6 µM; rhododendrin 26.3 µM; (+)-catechin 7-*O*-β-d-xylopyranoside 24.7 µM; and (+)-catechin 35.9 µM. Maximum concentrations of (+)-catechin 7-*O*-β-d-xylopyranoside or (+)-catechin were calculated assuming that either of them was the only one, as they co-eluted in HPLC. Impedance was recorded at 16 kHz every 60 s, continuously for up to 100 h.

### 3.8. Interferences of Optical Readouts by Natural Extracts

The NP1–NP4 extracts (0.5 mg/mL) were assayed for interference with three distinct cell viability assays. For the resazurin assay, each extract (50 μL) was incubated with 150 μL of resazurin (25 µM) for 2 h at 37 °C in 5% CO_2_ atmosphere. The fluorescence was measured at λ_excitation_ of 570 nm and λ_emission_ of 590 nm. The ATP assay was performed using the Promega’s CellTiter-Glo Cell Viability Assay. Each extract (100 μL) was incubated with the ATP reagent (100 μL), shaken for 2 min, followed by 10 min incubation at room temperature in darkness. The luminometric signal was recorded. For the LIVE/DEAD viability/cytotoxicity assay, 100 μL of each extract was incubated with 100 μL of calcein (2 μM) or EthD-1 (4 μM) in PBS. After 45 min, the fluorescence was recorded using λ_excitation_ of 485 nm and λ_emission_ of 530 nm (for calcein) and using λ_excitation_ of 530 nm and λ_emission_ of 630 nm (for EthD-1). All readouts were performed with a Varioskan Flash plate reader (Thermo Fisher Scientific, Vantaa, Finland).

### 3.9. Data Processing and Statistical Analysis

For the ECIS optimization, at least two wells per treatment were included while for the rest of the experiments, one well per treatment was included. Statistical parameters were calculated to assess the assay quality: screening window coefficient (*Z′*), signal-to-background (S/B) and signal-to-noise (S/N), with the aid of following equations [34,56]:

S/N = (*X*_S_ − *X*_B_)/(SD_S_^2^ + SD_B_^2^)^1/2^ (1)

S/B = *X*_S_/*X*_B_ (2)

*Z′* = 1 − [(3 × SD_S_ + 3 × SD_B_)/*X*_S_ − *X*_B_] (3)

Maximal signal (*X*_S_) was recorded in the electrodes covered by untreated cells at 24 or 48 h after the cells were added into the electrodes, while minimal signal (*X*_B_) was measured in electrodes where only culture medium was added, at the same time points. SD_S_ and SD_B_ correspond to the standard deviations of the maximal (*X*_S_) and the minimal (*X*_B_) signals calculated from four replicate wells. For the statistical analysis of the results presented in Table 2, the unpaired *t*-test (two-tailed) with Welch’s correction was applied, using Prism 5 for Mac OS X (GraphPad Software, La Jolla, CA, USA). *p* < 0.05 was considered statistically significant.

## 4. Conclusions

We have developed ECIS as a method for the assessment of cytotoxicity caused by natural products, with very noticeable advantages: (i) it is non-invasive, requires minimal labor, minute consumption of extracts, and/or natural pure compounds; (ii) it can be used for samples (both pure compound and multicomponent samples) that interfere with label-dependent assays; (iii) it allows detecting of acute and delayed cytotoxicity within a single run; and (iv) it enables continuous readouts, thus providing mechanistic insights of time-dependent cellular events triggered by natural products.

Based on these elements, we believe that ECIS represents a very suitable functional screening assay applicable to toxicological evaluations of herbal drugs, plant or microbial extracts, as well as natural pure compounds. It is a useful bioactivity-tracking tool that can facilitate and make the work with natural products less challenging within the drug discovery workflow.

## Supplementary Materials

Supplementary materials can be found at http://www.mdpi.com/1422-0067/16/11/26014/s1.
