# Supplementary material for: Online Measurement of Real-Time Cytotoxic Responses Induced by Multi-Component Matrices, such as Natural Products, through Electric Cell-Substrate Impedance Sensing (ECIS)
Source: Int J Mol Sci. 2015 Nov 12;16(11):27044–57. doi: 10.3390/ijms161126014 (PMC4661872; doi:10.3390/ijms161126014)
Supplement: Supplementary file 1 [file ijms-16-26014-s001.pdf]

Supplementary Materials: Online Measurement of Real-Time Cytotoxic Responses Induced by Multi-Component Matrices, such as Natural Products, through Electric Cell-Substrate Impedance Sensing (ECIS)

Adyary Fallarero, Ana E. Batista-González, Anna K. Hiltunen, Jaana Liimatainen, Maarit Karonen and Pia M. Vuorela

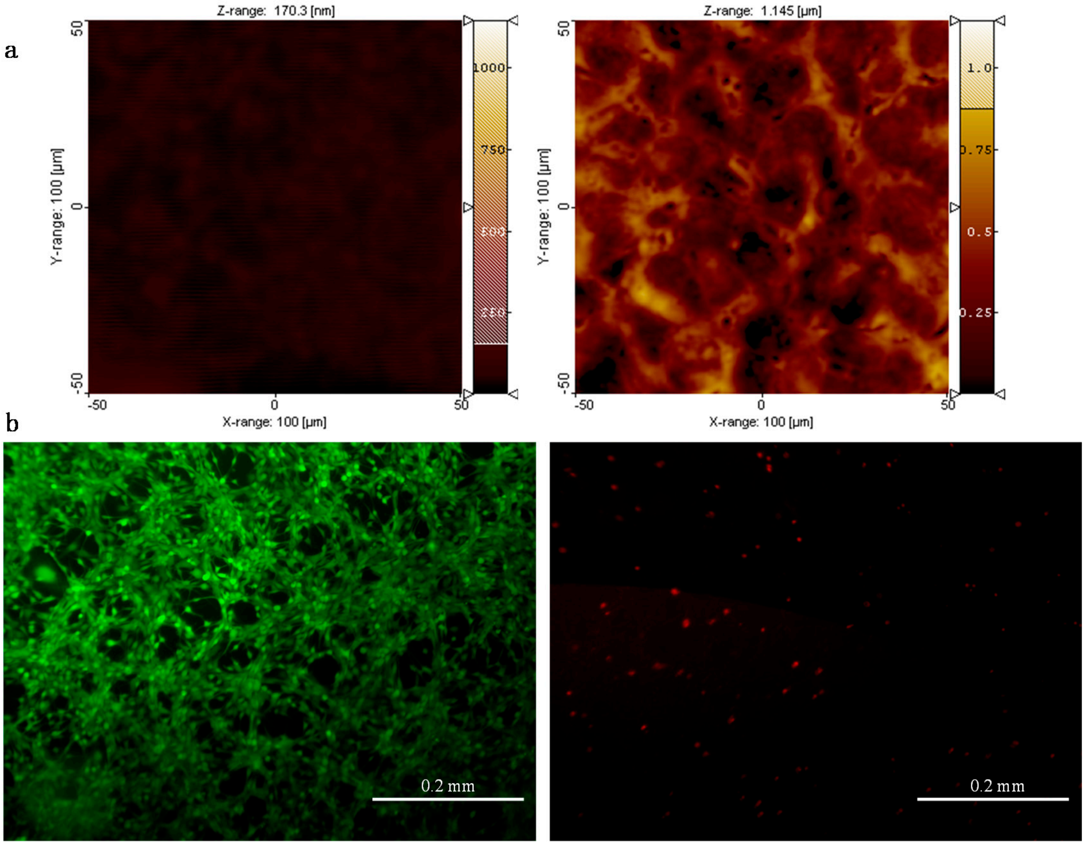

Figure S1. AFM (atomic force microscopy) images of the culture media samples (**left**) and untreated cells (**right**) added in the uncoated ECIS (Electric Cell-substrate Impedance Sensing) electrodes after 48 h (**a**); Fluorescence microscope images of calcein-stained alive (**left**) and EthD-1-stained (ethidium homodimer-1) dead (**right**) untreated cells grown for 48 h on the ECIS electrodes. Metabolically active cells and cells with damaged membranes emit green and red fluorescence, respectively (**b**). In (**b**), scale bars are 0.2 mm. In both cases, suspensions of cells (4×10^5 cells/mL) were used.
